# The Effect of Hygiene-Based Lymphedema Management in Lymphatic Filariasis-Endemic Areas: A Systematic Review and Meta-analysis

**DOI:** 10.1371/journal.pntd.0004171

**Published:** 2015-10-23

**Authors:** Meredith E. Stocks, Matthew C. Freeman, David G. Addiss

**Affiliations:** 1 Children Without Worms, Task Force for Global Health, Decatur, Georgia, United States of America; 2 Department of Environmental Health, Rollins School of Public Health, Emory University, Atlanta, Georgia, United States of America; Michigan State University, UNITED STATES

## Abstract

**Background:**

Lymphedema of the leg and its advanced form, known as elephantiasis, are significant causes of disability and morbidity in areas endemic for lymphatic filariasis (LF), with an estimated 14 million persons affected worldwide. The twin goals of the World Health Organization’s Global Program to Eliminate Lymphatic Filariasis include interrupting transmission of the parasitic worms that cause LF and providing care to persons who suffer from its clinical manifestations, including lymphedema—so-called morbidity management and disability prevention (MMDP). Scaling up of MMDP has been slow, in part because of a lack of consensus about the effectiveness of recommended hygiene-based interventions for clinical lymphedema.

**Methods and Findings:**

We conducted a systemic review and meta-analyses to estimate the effectiveness of hygiene-based interventions on LF-related lymphedema. We systematically searched PubMed, Embase, ISI Web of Knowledge, MedCarib, Lilacs, REPIDISCA, DESASTRES, and African Index Medicus databases through March 23, 2015 with no restriction on year of publication. Studies were eligible for inclusion if they (1) were conducted in an area endemic for LF, (2) involved hygiene-based interventions to manage lymphedema, and (3) assessed lymphedema-related morbidity. For clinical outcomes for which three or more studies assessed comparable interventions for lymphedema, we conducted random-effects meta-analyses. Twenty-two studies met the inclusion criteria and two meta-analyses were possible. To evaluate study quality, we developed a set of criteria derived from the GRADE methodology. Publication bias was assessed using funnel plots. Participation in hygiene-based lymphedema management was associated with a lower incidence of acute dermatolymphagioadenitis (ADLA), (Odds Ratio 0.32, 95% CI 0.25–0.40), as well as with a decreased percentage of patients reporting at least one episode of ADLA during follow-up (OR 0.29, 95% CI 0.12–0.47). Limitations included high heterogeneity across studies and variation in components of lymphedema management.

**Conclusions:**

Available evidence strongly supports the effectiveness of hygiene-based lymphedema management in LF-endemic areas. Despite the aforementioned limitations, these findings highlight the potential to significantly reduce LF-associated morbidity and disability as well as the need to develop standardized approaches to MMDP in LF-endemic areas.

## Author Summary

The tropical disease lymphatic filariasis (LF) causes chronic swelling of the leg—lymphedema—in 14 million people worldwide. To stop the spread of LF, a program led by the World Health Organization (WHO) offers annual preventive drug treatment to affected communities. For people who already have lymphedema, WHO recommends simple hygiene-based measures that include skin care and limb movement. Yet only a small proportion of those with LF-related lymphedema have been trained in these measures. To determine the effectiveness of hygiene-based lymphedema management, we reviewed the scientific literature. Twenty-two studies were found that 1) used hygiene-based interventions to manage lymphedema; 2) measured the effect of these interventions; and 3) were done in an area where LF occurs. Overall, use of hygiene-based measures was associated with 60% lower odds of inflammatory episodes, known as “acute attacks,” in the affected limb. Acute attacks cause severe pain, fever, and disability, and they make lymphedema worse. Hygiene and sanitation are necessary for control of many tropical diseases. Hygiene is also effective for managing LF-related lymphedema and reducing suffering caused by acute attacks. Training people with lymphedema in hygiene-based interventions should be a priority for LF programs everywhere.

## Introduction

Lymphedema of the leg and its advanced form, known as elephantiasis, are significant causes of disability and morbidity in areas endemic for lymphatic filariasis (LF), with an estimated 14 million persons affected worldwide [1]. In 1998, the World Health Organization (WHO) launched its Global Program to Eliminate Lymphatic Filariasis (GPELF) with the twin goals of (a) interrupting transmission of the parasitic worms that cause LF and (b) providing care to persons who suffer from lymphedema and other clinical manifestations of LF [2, 3]. Although mass treatment with antiparasitic drugs has led to significant reductions in transmission worldwide [4], scaling up of LF morbidity management and disability prevention (MMDP) activities has lagged behind [5, 6]. Consequently, countries that have successfully interrupted transmission may have sizeable numbers of people who continue to suffer from the disabling and stigmatizing effects of lymphedema and elephantiasis [7].

Although the factors associated with progression of lymphedema in filariasis-endemic areas are complex, the importance of repeated episodes of acute bacterial dermatolymphangioadenitis (ADLA) has been demonstrated in several studies [8–11]. These inflammatory episodes, which are characterized by pain, fever, and swelling of the affected limb [12], further erode lymphatic function, stimulate fibrosis, and increase the risk of further ADLA episodes. Clinical studies suggest that a simple intervention to reduce ALDA risk, based on hygiene and skin care, can halt and even reverse this progression [13–18]. The intervention package goes by different names, e.g., “foot care” or basic lymphedema management, and includes various components based on resources (e.g., compressive bandaging) and local healing practices (e.g., yogic breathing), but hygiene (regular, careful washing with soap and water) is the central component in all of these packages. The importance of water, sanitation, and hygiene (WASH) is increasingly recognized as essential for the control and elimination of several neglected tropical diseases (NTDs), and systematic reviews have been recently published on the effect of WASH interventions on trachoma, soil-transmitted helminth infection, and schistosomiasis [19–21].

The reasons for the lagging promotion of MMDP are complex [6, 7] and include a lack of funding, inadequate integration of LF elimination into national health services, and the fact that relatively few LF program managers have been clinically trained. In addition, lack of consensus about the degree of effectiveness of WASH interventions on LF-related lymphedema is a contributing factor. To address this gap, we conducted a systematic review and meta-analysis of the literature to assess the evidence base and quantify the relationship between lymphedema management programs involving a hygiene component and health-related outcomes.

## Methods

### Search strategy

We performed a systematic review and meta-analysis of the literature to address the effects of hygiene-based interventions on morbidity and quality of life related to lymphedema in filariasis-endemic areas. We systematically searched PubMed, Embase, ISI Web of Knowledge, MedCarib, Lilacs, REPIDISCA, DESASTRES, and African Index Medicus databases with no restrictions on language or year of publication. Our search was performed through March 23, 2015 with no restriction of start date. We employed a broad set of search terms, pairing the terms [lymphedema] and [lymphoedema] with the following keywords: [water], [hygiene], [hand wash*], [foot wash*], [soap], [morbidity management], [morbidity control], [disability prevention]. In addition, we hand-searched the bibliographies of relevant publications. Any additional articles found to be pertinent during this process were included.

Studies were included in the systematic review if they 1) were conducted in an area endemic for lymphatic filariasis as determined by WHO (5); 2) involved lymphedema management that included hygiene; and 3) assessed lymphedema-related morbidity (e.g. incidence of ADLA, change in limb circumference) or quality of life. All studies were eligible if they met these inclusion criteria; however, because prophylactic antibiotics have been shown to reduce incidence of ADLA, any study arms that included prophylactic antibiotics were excluded from the meta-analysis. If an article was considered relevant but data were not available in the format needed for our meta-analysis, the corresponding authors were contacted by e-mail and asked to supply the relevant data. We conducted meta-analyses for specific exposure-outcome relationships based on available data. Meta-analyses were conducted in adherence to the PRISMA statement (S1 Text). Our complete protocol is available in the supplemental materials (S2 Text).

### Search strategy, selection criteria and data extraction

Articles were selected for inclusion using a two-step review process. First, the titles and abstracts of all identified studies were examined, and studies that failed to meet the inclusion criteria after this step were excluded. Second, two reviewers (M.E.S. and M.P.) independently examined the full text of potentially relevant articles using a standard protocol developed by M.E.S. and M.C.F. In the event of disagreement regarding the eligibility of a study during this phase, the opinion of a third reviewer (D.G.A.) was sought, and the parameters of the study’s inclusion were discussed until consensus was reached.

Once a set of eligible studies was agreed upon, relevant data were extracted from each study by M.E.S. using a standard protocol. To ensure extraction reliability, M.P. also extracted data from a subset of 10% of identified studies, and no discrepancies were found. Data extracted included a brief description of the study (e.g., study design, setting, year, and sample size), details of the study population, a description of the components of lymphedema management, details about how morbidity was measured, and study results. Measures of lymphedema-related morbidity included incidence of acute attacks (ADLA), limb volume or circumference, days of work missed due to disability, and other variables. Clinical signs of morbidity were measured by researchers through physical examination and interviews. Physical examinations commonly focused on staging of the limbs for lymphedema severity and the presence of skin lesions that could serve as entry points for infection, usually on the feet and between the toes. Quality of life was assessed by questionnaire.

### Quality issues

To determine the quality of identified studies, we developed a set of criteria derived from the GRADE methodology. Our criteria took into account diagnostic features, characteristics of the lymphedema management programs, study design, and overall strengths and limitations of the studies. Studies were awarded 1 point for meeting each individual criterion listed in Table 1 and could obtain an overall score ranging between 0 and 10 points for each meta-analysis. M.E.S. performed the quality assessment independently and documented the results (S3 Text). All relevant studies were included in the review regardless of their overall quality rating. Quality ratings did not affect the summary of effect measures, but they help to demonstrate the overall quality of individual studies and to identify research gaps.

**Table 1:** Criteria for which studies received +1 point for GRADE score.

**Morbidity Assessment**
** Research team clearly defined how lymphedema patients were identified (e.g. recruited from LF care facility)**
** Lymphedema staged using standard criteria, which were specified**
** Morbidity outcomes assessed directly by research team (e.g. leg volume measured, ADLA episodes observed)**
** Subjects monitored and morbidity assessed prospectively (e.g. research team visited homes of patients monthly)**
**Morbidity Management**
** Hygiene component clearly described**
** Hygiene specifically emphasized as key component of intervention**
** Research team ensured patients had materials needed for hygiene (e.g. checked presence of, or provided soap, water, wash basins)**
** Research team attempted to verify compliance with recommended interventions (e.g., through home visits, educational refreshers, etc.)**
**Study Design**
** Control group(s) present**
** Analysis attempted to control for possible confounders**

### Meta-analysis

We conducted meta-analyses when three or more studies reported the same measure of effect on morbidity (e.g., ADLA incidence). For studies with multiple arms that included prophylactic antibiotics, only the arm that did not include prophylactic antibiotics was used in the analysis. Observation periods for ADLA varied among studies. In the meta-analysis of ADLA incidence, the observation period was standardized to person-year. For the analysis of change in the proportion of patients with one or more ADLA episodes in a given observation period (both before and after lymphedema management was initiated) it was not possible to standardize the observation period. Findings of studies included in the systematic review, but not meeting the criteria for a meta-analysis were summarized and examined for patterns, as recommended by the Cochrane Review [22].

We used Microsoft Excel (Microsoft Corporation, Redmond, WA, United States of America) to conduct meta-analyses and to develop forest plots [23]. Funnel plots were utilized to investigate the existence of publication bias [24]. Heterogeneity between studies was determined using Higgins’ I^2^ and Cochran’s Q-tests [22]. Random effects models were used throughout to enhance generalizability of results [25], and pooled estimates for the effect of lymphedema management on measures of morbidity were employed [22].

## Results

### Characteristics of identified studies

**Fig 1:**
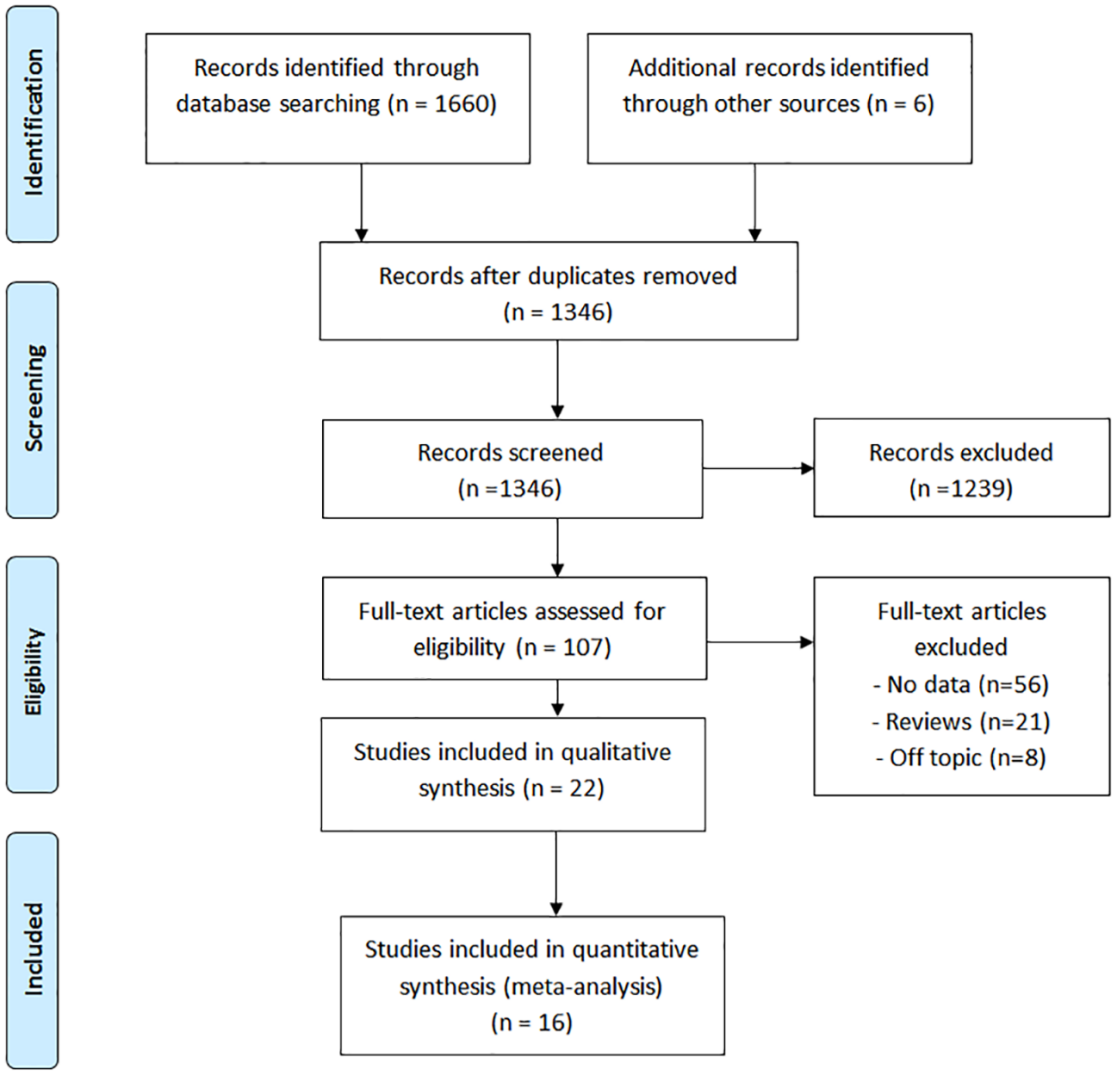
Flow chart of publications identified and excluded for this review.

**Fig 2:**
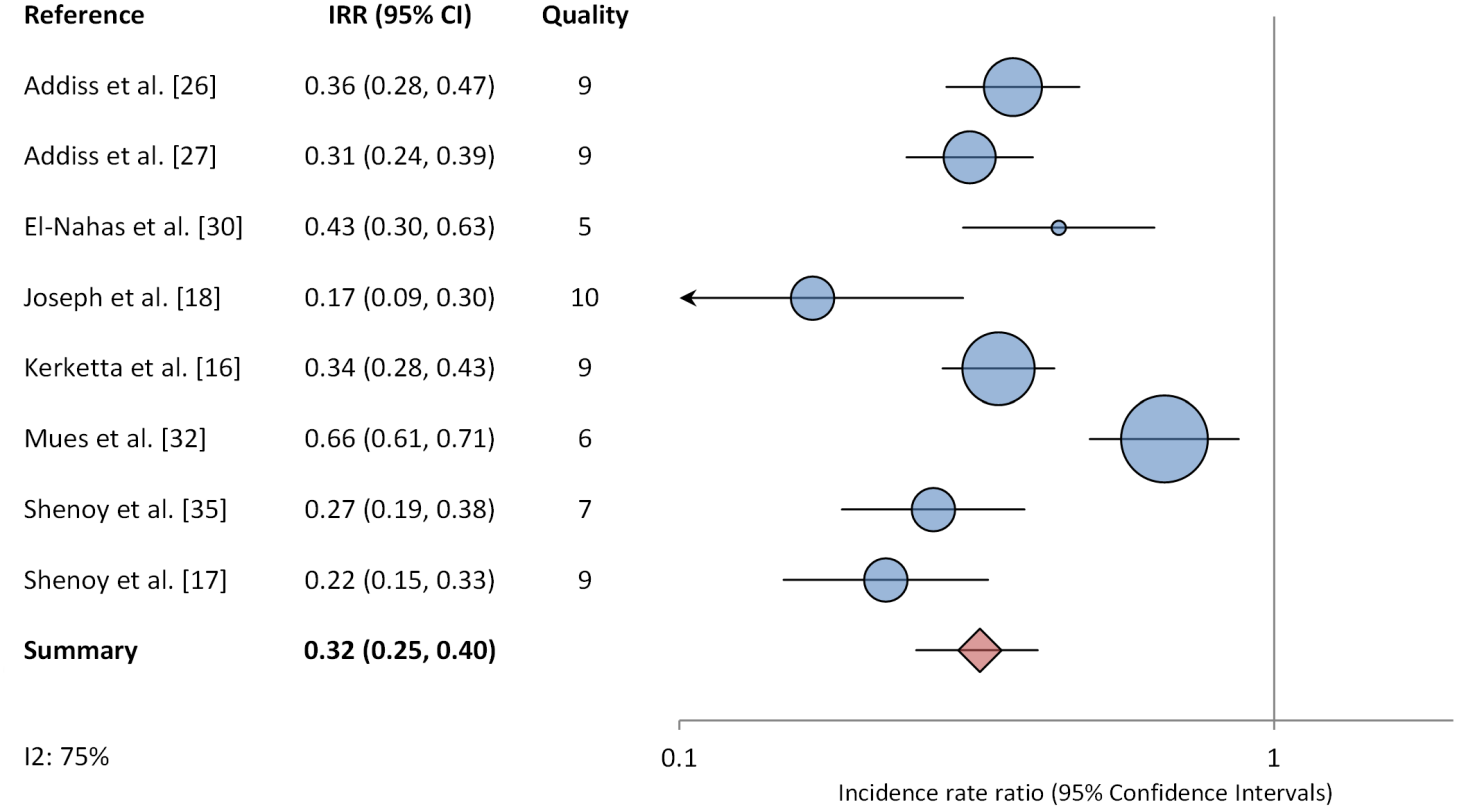
Meta-analysis examining the association between hygiene-based lymphedema management and the incidence of acute dermatolymphangioadenitis ADLA).

**Fig 3:**
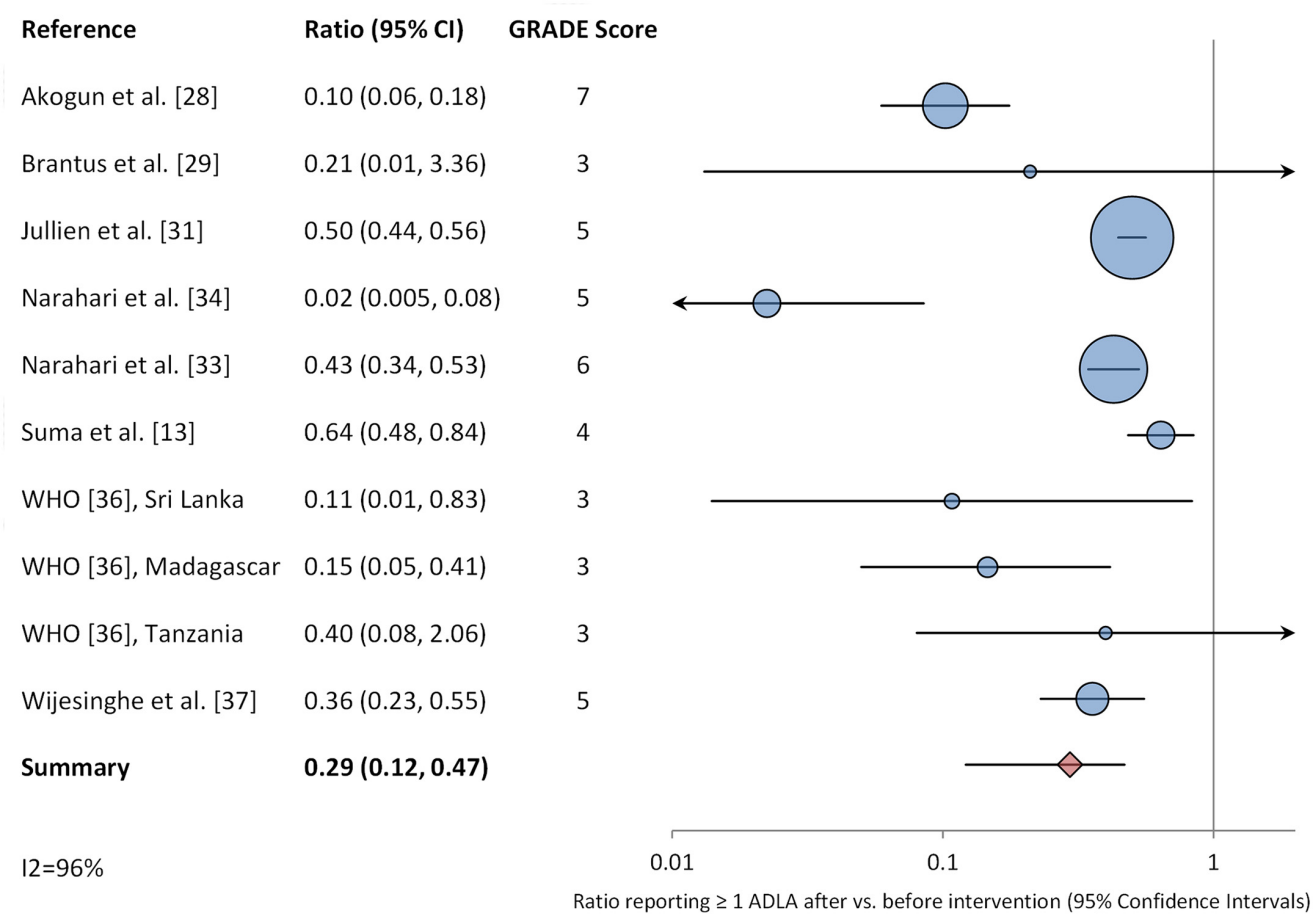
Meta-analysis examining the association between hygiene-based lymphedema management and the percentage of patients reporting at least one episode of ADLA during a specific time interval.

Our initial search yielded 1,666 publications (Fig 1). One hundred seven publications were deemed relevant after review of titles and, when available, abstracts. These articles were fully screened by M.E.S. and M.P. Following this screening, 22 articles were determined to meet systematic review inclusion criteria (Table 2) [13, 14, 16–18, 26–42]. We conducted two meta-analyses. One included eight studies reporting ADLA incidence ratios (Fig 2). The other included eight studies comparing the percentage of patients reporting at least one ADLA episode in a given time period before and after implementation of lymphedema management (Fig 3). Twenty of the included publications were intervention studies that involved a hygiene education component; the two remaining studies were cross-sectional studies comparing patients who had and had not participated in a hygiene-based intervention program. In three studies, antibiotics were administered in response to acute attacks. Five additional studies had study arms in which antibiotics were administered prophylactically at the time of health education; as noted earlier, these study arms were not included in the meta-analyses. Seven studies (32%) were conducted in Africa, 12 studies (55%) were conducted in Asia (primarily India and Sri Lanka) and three studies (14%) were conducted in the Americas. The quality scores of studies included in the meta-analyses were generally moderate to high (S3 Text).

**Table 2:** Summary of studies reporting on lymphedema morbidity management program.

Reference	Study Design & Setting	Timeframe	Study Population	Description of Morbidity Management Program	Measure of Morbidity	Data Obtained
**Addiss, et al., 2011 [** 27 **]**	Randomized double-blinded clinical trial at clinic at Ste. Croix Hospital in Leogane, Haiti, comparing regular and antibacterial soap used in foot-care.	12 months (beginning in Spring 2001)	197 patients at the lymphedema clinic who lived within 10 km of hospital and were competent in lymphedema self-care.	Clinic-based research staff supplied patients with soap and made monthly visits to patients' home or workplace to resupply soap and monitor compliance.	Self-reported ADLA in previous month. Staff also assessed stage of affected limbs and checked for lesions.	ADLA incidence per person-year, incidence rate ratio (95% CI), comparing before and after entry into the study
**Addiss et al., 2010 [** 26 **]**	Prospective, two phase intervention study in Leogane, Haiti. Phase I focused on reducing leg volume and included compressive bandaging and a component of "complex decongestive physiotherapy"; Phase II primarily focused on preventing ADLA through hygiene and skin care.	1995–1998	175 people with lymphedema of the leg who enrolled in a lymphedema clinic in Leogane, Haiti	Patients taught to wash legs, apply antifungal and antibiotic creams, elevate limb, and perform range of motion exercises in March 1997 (Phase II, after a phase emphasizing compression bandaging). In Nov 1997, booklet with basic management messages given to each patient, and "soap opera" with a character implementing the management was broadcast over radio. Compliance measured in interviews.	Leg volume assessed using water displacement, classified according to stage, and patient asked about episode(s) of ADLA in past 12 months. Follow up every 4–6 weeks, where patients were asked to report any ADLA for which they hadn’t gone to the clinic.	Change in ADLA incidence (episodes per person-year); change in leg volume
**Aggithaya et al., 2013 [** 38 **]**	Intervention study in three endemic districts of Kerala province, India.	6 months	446 LF patients who attended a community LF morbidity management day camp.	Self-care integrative treatment camps within community where LF patients trained in skin care and daily yoga/breathing practices. Individual meeting with patients to ensure understanding. One month follow-up over phone to check compliance, final follow-up at six months.	Quality of life (QoL) of LF patients determined using validated and pretested specific questionnaire (LF-specific QoL questionnaire-LFSQQ). Disease burden assessed by asking questions about history of acute attacks and other symptoms.	Change in quality of life (reported as change in mean score on the questionnaire between baseline and 6-month follow-up)
**Akogun et al., 2011 [** 28 **]**	Intervention study in North-eastern Nigeria comparing three methods of LF care education: community care (CC), patient care (PC), and health facility care (HC).	12 months	Community members with previous experience with filarial episodic attack were asked to register at the health facility nearest them (registration remained open for the duration of the study).	CC arm selected one member to attend training and be responsible for educating people with LF in community. Training included identification of lymphoedema, basic hygiene procedures for management of lymphoedema and ADLA. In PC arm, small groups met in health facility. One leader chosen to attend LF care training and then train rest of group. In HC arm, patients attended clinic to be individually trained by staff and returned for checkups every third day. All received buckets, bowls, wash soap, towels, ointment.	Participants provided data on history of disease progression from onset of acute signs to development of chronic signs. Healthcare worker examined their limbs and recorded degree of morbidity (lesions on skin) and graded limbs according to the level of swelling and appearance of skin.	Change in mean ADLA frequency, change in duration of ADLA
**Brantus et al., 2009 [** 29 **]**	Education intervention pilot program, with follow-up once a month for 20 months to collect data on self-reported ADLA in previous month.	20 months	32 lymphedema patients living in Zanzibar, Tanzania.	Hygiene education to carefully wash affected limb and treatment of any wounds, regular exercise, elevation of affected limb, use of suitable footwear.	Self-reported incidence of acute attacks in previous month.	Reported ADLA incidence in previous month (reported for 20 months)
**Budge et al., 2013 [** 39 **]**	Prospective cohort study in 30 villages in Orissa State, India	24 months (2009–2011)	370 patients at least 14 years of age from 30 villages who reported lymphedema lasting more than three months (identified from house-to-house morbidity census).	Indian NGO, Church's Auxiliary for Social Action (CASA) provided community-based treatment of lymphedema using a network of village volunteers who are trained to provide home-based care and instruction in lymphedema management techniques and use of hygiene supplies.	Independent staging of leg(s), photographs taken, staff used 7-stage Dreyer system. ADLA episodes reported by patient for previous 30 days. Patients also given the WHO Disability Assessment Schedule II at baseline (July 2009) and regular intervals (1, 3, 6, 12, 18, 24 months, through July 2011) to assess patients' perceived disability.	Change in perceived disability (WHO Disability Schedule II), change in reported ADLA incidence in previous 30 days, change in stage of most-affected leg, days of work lost
**El-Nahas et al., 2011 [** 30 **]**	Intervention study for lymphedema management package in Egypt.	24 months (2008–2010)	45 patients attending Mansoura University Hospitals complaining of limb swelling with present or past history of limb redness.	Patients trained for basic lymphedema management, including daily care of affected limb, treatment of wounds with topical antibiotics, treatment of fungal infections with topical antifungal, manual draining, massage, use of compression, and elevation.	Self-reported incidence of acute attacks in previous year (pre-treatment year vs. post-treatment year).	Reported ADLA incidence in previous year
**Harvey et. al., 2011 [** 40 **]**	Retrospective analysis of annually collected data from Togo after implementation of a National Lymphoedema Management Programme (NLMP)	2007–2010	Survey cohort was convenience sample of 166 people with lymphedema, with same individuals followed each year.	Togo’s NLMP (began in 2007) teaches lymphoedema patients management techniques in order to improve self-care behavior and outcomes among patients.	Lymphedema-related symptoms were ascertained from the interview. Quality of life questions were asked, and patients were scored on the Duke Anxiety-Depression (DUKE-AD) scale.	Change in ADLA incidence, change in depression levels
**Joseph et al., 2004 [** 18 **]**	Double-blind, placebo-controlled, clinical study for antibiotic treatment with hygiene education in 22 villages in Vellore district, Southern India	12 months treatment, 12 months follow-up	Screened 430 villagers, accepted 150 subjects who were >15 years, weighed >30 kg, and had experienced at least two ADLA attacks in the preceding year.	Training period before treatment where patients taught about hygienic care of limb, stressing four main components: periodic nail clipping, nightly cleansing of affected area with soap and water, importance of keeping limb dry between washes, and application of salicylic acid ointment to skin (between toes and on sides of feet).	Field workers visited subjects at home every 3 to 4 days during 12 month follow-up during treatment and for following 12 months. Monitored treatment adherence, incidence of ADLA attacks, and any adverse effects of treatment. Also some data on mean volumes of affected limbs, grade, serology.	Change in incidence of ADLA attacks (mean attacks per person-year), change in average limb volume.
**Jullien et al., 2011 [** 31 **]**	Intervention study for home-based lymphoedema management programme in primary health care system of Burkina Faso	4.5 months	1089 patients suffering from LF-related lymphoedema of leg at any stage who participated in health education and washing project between April 2005 and December 2007.	Health education and washing project which included hygiene, washing, teaching good practices, and treatment of wounds, acute attacks, and other ailments.	Interviewed during monthly consultations at the health facility about acute attacks in preceding month	Change in percentage of patients reporting and/or caregivers observing ADLA in previous month
**Kerketta et al., 2005 [** 16 **]**	Intervention study of footcare with or without antibiotics in eight randomly selected villages in Khurda district of Orissa, India with follow up every two weeks.	12 months	254 patients identified through house-to-house visits in eight randomly selected villages.	Three arms of the study: (1) penicillin + footcare, (2) diethylcarbamazine (DEC) + footcare, (3) footcare + topical antiseptic ointment.	ADLA history reported by recall every two weeks.	Mean ADLA frequency before and after treatment
**Mathieu et. al., 2013 [** 42 **]**	National Lymphedema Management Programme in Togo.	24 months	109 patients in randomly-selected villages used in analysis for ADLA	Self-care techniques, including regular washing of the leg with soap and water, drying, elevating the affected limb, and regular exercise were demonstrated to patients by trained health workers. Patients were also provided with an educational booklet with illustrations.	Interview about acute attacks in preceding year	Change in incidence of ADLA attacks, comparing pre-intervention and follow-up periods
**McPherson et al., 2003 [** 14 **]**	Clinical intervention study in Wismar, Guyana.	April—Jun 2001	14 community members suffering from lymphedema	Each patient individually educated on importance of hygiene, skin care, and elevation, as well as simple exercises. Appropriate treatment given at start (antibiotics, antiseptics, and topical creams) and whenever necessary throughout.	Diagnosis confirmed clinically and classified according to Dreyer staging criteria. Interview about disease history and frequency of acute attacks. Also asked questions regarding knowledge, attitudes, and practices (KAP) and completed dermatology quality of life index (DQLI).	Change in DLQI score
**Mues et al., 2014 [** 32 **]**	Lymphedema management program implemented in Odisha State, India from 2007–2010 by Church's Auxiliary for Social Action (CASA) in consultation with CDC	24 months	370 lymphedema patients >14 years old reporting leg swelling for at least 3 months, from 30 villages that had not been enrolled in the program and were not in immediate vicinity of a participating town.	Patients trained in basic lymphedema management by physician-trained volunteers, including daily washing of limbs with soap and water, exercise and elevation of affected limb, and use of footwear outside. Patients trained in importance of early treatment and prevention of secondary infections and supplied with 6 months’ of soap and antifungal cream.	Self-reported ADLA episodes in previous month, defined as presence of two or more of following symptoms: redness, pain, or swelling of the leg or foot, with or without the presence of fever or chills.	Change in incidence of ADLA attacks
**Narahari et al., 2013 [** 33 **]**	Non-randomized interventional study in two LF endemic districts in southern India.	3.5 months	730 patients (851 affected limbs) known to live in area of study. Only those with stage 2 or 3 lymphedema were enrolled.	All patients given training in integrative management which involved patient education and self-care using a domiciliary protocol.	Patient limbs graded in camp, thigh volume assessed, information gathered on inflammatory episodes and skin lesions. Quality of life assessed using an LF-specific questionnaire.	Quality of life, limb volume before and after, number and percentage of patients reporting ADLA in previous 3 months.
**Narahari et. al., 2007 [** 34 **]**	Intervention study of "reverse pharmacology design" in India.	194 days of treatment over 3 years (2003–2006)	240 patients having lymphedema of one or both lower limbs with ability to withstand a variety of yoga exercises.	Ayurveda, yoga, and biomedicine components applied together: soap wash, soaking affected limb, Indian manual lymph drainage (IMLD), yoga, compression, dietary restrictions, oral herbal medication for elephantiasis.	Limb circumference at various locations, limb volume by water displacement.	Change in limb volume and circumference, "history of inflammatory episodes"
**Shenoy et al., 1998 [** 35 **]**	Double-blind, placebo-controlled, clinical study comparing efficacy of local treatment of affected limb combined with repeated doses of ivermectin, DEC, or placebo.	24 months	120 patients attending filariasis clinic who had experienced at least 2 ADLA attacks in the past year.	Patients in all arms of study instructed on local care and hygiene of affected limbs.	Reported ADLA attacks in previous year.	Total reported ADLA attacks in the year before and after treatment.
**Shenoy et al., 1999 [** 17 **]**	Randomized control trial for antibiotics and footcare (including antibiotic cream) in Alappuzha, India.	12 months treatment, 12 months follow-up	Patients attending clinic of the Filariasis Chemotherapy Unit of the T.D. Medical College Hospital who had underlying filarial oedema and had experienced at least 2 attacks of ADLA in past 12 months.	All patients were asked to clean limbs with soap and water every night, keep affected limbs dry, clip nails, and apply salicylic acid ointment between toes, on nails, and on sides of feet each night. Overall compliance checked at regular intervals using surprise checks and pill counts.	Pre-study, all patients hospitalized for 4 days. Initial examination and interview about previous attacks, then a year of study and a year of follow-up. Follow-up every two weeks plus any time there was an ADLA attack.	Change in ADLA incidence (mean no. attacks/year) from pre-treatment year to treatment year, to follow-up year.
**Suma et al., 2002 [** 13 **]**	Cross-sectional evaluation of patients one year following clinical trial for antibiotics and footcare programme in Alappuzha district, India	—	150 patients who participated in antibiotics/footcare clinical trial >1 year ago (see Shenoy et al., 1999).	Unsupervised, post-intervention foot care included cleaning affected limb every night with soap and water, keeping limb dry, applying salicylic acid ointment to webs of toes, nails, and sides of feet each night, clipping nails regularly, and encouraging regular use of footwear.	Medical officer asked about footcare procedures in the unsupervised period, as well as the occurrence of ADLA attacks in the previous year (number, intensity, duration, precipitating factors, treatment received). Also examined and graded lymphoedema and checked skin condition.	Change in ADLA incidence, comparing pre-intervention and follow-up periods
**WHO, 2004 [** 36 **]**	Three pilot projects on washing affected limbs in Madagascar, Sri Lanka and Zanzibar.	2004	n/a	Washing affected limb daily, with home visits by volunteer health workers.	Self-reported acute attacks in previous month.	Reduction in ADLA attacks in previous month; change in percentage of sufferers reporting at least one acute attack in previous month.
**Wijesinghe et. al., 2007 [** 37 **]**	Quasi-experimental, interventional study with a pre-test/post-test design, carried out over 18 months in Colombo, Sri Lanka.	18 months in 2004–2005	Consecutive sample of 163 patients with lymphoedema attending two filariasis clinics in Colombo, Sri Lanka.	Principal investigator individually taught each patient the WHO-recommended comprehensive regime of limb care, emphasizing washing limbs with soap and water, especially toe webs and skin folds, keeping clean limbs dry, elevating limbs, and exercising affected limbs. Provided WHO booklet with explanatory pictures and text.	Lymphedema diagnosed in clinic (non-filarial causes excluded) and graded according to WHO recommendations, then interviewed about ADLA suffered in the last year (number, duration and intensity of attacks, and details of any precipitating factors, management measures taken, and treatment received). Patients asked to come into hospital monthly for any symptoms and to follow-up one year after initial training. Attacks treated with oral antibiotics/creams. Post-intervention assessment at 12 months.	Change in percentage of patients reporting ADLA in past year, change in duration of ADLA, change in frequency of ADLA, change in grade, change in compliance with washing.
**Wilson et al., 2004 [** 41 **]**	Intervention study evaluating the effect of lymphedema management on histologic features of skin punch biopsies in Leogane, Haiti	12 months	91 patients living <10 miles from the lymphedema treatment clinic who reported no ADLA episodes in previous 2 weeks.	Patients instructed in lymphedema self-care, with emphasis on daily washing, basic skin care to treat/prevent entry lesions, range of motion exercises, and elevation of limb.	Patients tested for filarial infection and lymphedema stage was assessed using an adaptation of a three-stage system recommended by WHO. Punch biopsies obtained at beginning of treatment and approximately 1 year later	Change in histologic features on punch biopsy

### Components of lymphedema management programs

Lymphedema management programs all included a hygiene element, usually in the form of education about the importance of daily washing the affected limbs with soap and water. The health education intervention varied from study to study (Table 1), but generally involved training by a nurse or other healthcare provider. In 10 (45%) studies, soap was provided to the patients to ensure its availability when washing limbs. Eight studies also emphasized yoga or other movement or exercises, two studies included manual lymph drainage of the affected limb(s), and three studies used compression bandaging. Only seven (32%) studies explicitly stated that they checked for compliance with limb care (through soap checks or questionnaires), while 12 (55%) studies provided antiseptic or antibiotic foot creams to supplement foot washing with soap and water.

### Measures of morbidity and quality of life

The most commonly reported outcome measures are summarized in Table 3; they included various measures of ADLA (n = 18); perceived quality of life, disability, or depression (n = 5); limb circumference and/or volume (n = 3); stage or grade of affected limb (n = 4); days of work lost due to disability (n = 1); and changes in histologic features in the affected limb(s) (n = 1).

**Table 3:** Studies reporting on various measures of lymphedema-related morbidity.

Outcome measure	No. of studies	References
**Change in ADLA incidence**	13	[13, 16–18, 26, 27, 29, 30, 32, 34, 35, 40, 42]
**Change in patients reporting at least one ADLA attack/time period**	8	[13, 28, 29, 31, 33, 34, 36, 37]
**Change in reported quality of life measure** *	5	[14, 33, 38–40]
**Change in stage or grade of affected limb**	4	[32, 37, 39, 41]
**Change in leg volume**	3	[26, 33, 34]
**Change in limb circumference**	2	[34]
**Change in duration of acute attack**	2	[28, 37]
**Change in histology of skin punch biopsy**	1	[41]
**Change in days lost of work**	1	[13]

*Defined and measured in various ways across studies, including perceived quality of life, disability, and depression.

### ADLA incidence and duration

Of the 18 studies reporting some measure of ADLA, 8 (42%) provided enough data to calculate ADLA incidence ratios and were included in a random-effects meta-analysis, which provided a pooled estimated IRR of 0.32 (a mean of 2.39 and 0.69 episodes per person-year before and after intervention, respectively), with a 95% confidence interval 0.25–0.40 (Fig 2). Heterogeneity was fairly high (I^2^ = 75%).

Eight publications (including one report from the World Health Organization with data for three separate pilot projects [36]) compared the percentage of study participants reporting at least one ADLA in an equivalent time period before and at some time after implementation of the lymphedema management intervention. The periods of observation or recall were 1 month [29, 31, 36], 3 months [33], 1 year [13, 28, 37], and unspecified [34]. These were included in a random-effects meta-analysis. The proportion of lymphedema patients experiencing at least one ADLA episode in a given time period decreased from 49.6% at baseline to 16.2% after implementation of lymphedema management, with a pooled estimated ratio of 0.29 and a 95% confidence interval of 0.12–0.47 (Fig 3). Heterogeneity was high (I^2^ = 96%).

Change in duration of acute attacks was reported in two studies. Akogun and colleagues found that duration of acute attacks declined with consistent foot care in all intervention arms, but most noticeably between the first and twelfth months [28]. Wijesinghe et al. found that the mean duration of acute attacks was slightly shorter after implementation of lymphedema management than at baseline (5.70 days vs. 5.84 days, p>0.05) [37].

### Quality of life, depression, and perceived disability

Five studies reported on some measure of quality of life or emotional wellbeing. No meta-analysis was conducted because the tools and methods used to quantify quality of life varied across studies.

#### Quality of life

Aggithaya and colleagues used an LF-specific quality of life questionnaire (LFSQQ) to assess perceived quality of life of patients before and after participation in basic lymphedema management and found that overall quality of life increased significantly [38]. Narahari et al., also found statistically significant improvements in quality of life scores on LFSQQ among patients who attended an LF training “camp” [33].

#### Disability

Budge et al. administered the WHO Disability Assessment Schedule II at regular intervals to 370 lymphedema patients in order to assess perceived disability due to lymphedema. Disability scores decreased significantly (p<0.0001) in patients enrolled in a community-based lymphedema management program, particularly among patients with moderate to advanced lymphedema [39]. Additionally, patients reported losing an average of 2.5 fewer work days per month (p<0.001) after the implementation of a community-based lymphedema management program [39]. A study from Togo found significant decreases in depression, based on the Duke Anxiety-Depression (DUKE-AD) scale, after the implementation of a National Lymphoedema Management Program [40]. After hygiene training and antifungal cream were provided to patients in Guyana, McPherson and colleagues found a significant mean improvement of 6.8 points (p<0.0001) on the Dermatology Life Quality Index (DLQI) scale, which is a dermatology-specific quality of life questionnaire [14].

### Limb circumference and volume

Three studies measured morbidity through limb circumference and/or volume. No meta-analysis was conducted because the methods used to measure leg circumference and/or volume varied across studies. Addiss et al. assessed leg volume using water displacement and found that 78.3% of patients experienced a reduction in leg volume after the implementation of basic lymphedema management emphasizing hygiene and self-care, especially among those with legs with more advanced lymphedema [26]. Narahari and colleagues found a statistically significant 1% reduction in thigh volume among patients who attended LF training “camps” [33], and in another study, found that both circumference and volume of affected limbs decreased significantly after implementation of a comprehensive lymphedema management program [34].

### Stage or grade of affected limb

Four studies reported on change in the stage or grade of the affected limb. No meta-analysis was conducted because the standardized staging systems used on affected limbs varied across studies. Budge et al. conducted independent staging of leg(s) using the 7-stage Dreyer system [43], and found that patients with moderate (stage 3) or advanced (stages 4–7) lymphedema in affected limbs experienced significantly greater improvement after implementing a community-based treatment program for lymphedema, which employed a network of village volunteers trained to provide home-based care and education in lymphedema management [39]. Wijesinghe et al. found that, after the intervention period, a significant number of patients had reductions from grade II to grade I lymphedema, according to the WHO-recommended lymphedema staging system [37, 44]. In another study using the WHO-recommended lymphedema staging system, Wilson et al. found no significant change in the stage of affected limbs [41].

### Other measures of morbidity

Wilson et al. examined changes in histologic features from skin-punch biopsies before and after training in lymphedema self-care, with an emphasis on hygiene, basic skin care, range of motion exercises, and limb elevation. Several histologic improvements were noted in the follow-up biopsies of the leg, most notably reductions in fibrosis [41].

## Discussion

The physical, social, and psychological suffering caused by lymphatic filariasis is enormous [45]. Our understanding of the nature and magnitude of this suffering is still incomplete, although several studies in LF-endemic areas have documented significant adverse impacts of lymphedema on physical and mental health, as well as on quality of life. Lymphedema patients frequently report depression, embarrassment, and increased social isolation [46, 47], even in the early stages of the disease [48]. The stigma of lymphedema creates barriers both to health-seeking and to adherence to recommended treatment [49]. Stigma also leads to social disconnectedness, which, in turn contributes to depression and other negative health outcomes [50]. In the absence of appropriate MMDP programs, misdiagnosis and improper treatment is not uncommon, and patients seek out cures and treatments that may be ineffective and expensive [51]. Additionally, increased frequency of ADLA may negatively impact socio-economic status through decreased ability to work and perhaps even reduced cognition [52].

Given these far-ranging consequences, providing treatment to the 14 million people who suffer from lymphedema in LF-endemic areas is an urgent matter, both for health and human dignity. Much of this suffering is related to ADLA [52]; thus, measures to reduce ADLA are especially important. The pioneering studies of Dreyer and colleagues in Brazil and Shenoy and colleagues in India provided the first convincing evidence of salutary effects of hygiene-based measures on ADLA [10, 12, 35] and established them as the cornerstone of the MMDP “pillar” of the GPELF [3, 6]. Despite this evidence and the development of WHO training modules on hygiene-based lymphedema management, uptake has been particularly slow. Although there are undoubtedly many factors involved, one of these has been a lack of recognition as to the degree to which these simple measures reduce ADLA and other morbidity.

Our meta-analyses revealed a significant association between hygiene-based interventions and decreased ADLA. Although meta-analyses for other physical measures of morbidity (e.g. stage of affected limb, limb circumference/volume) were not feasible, several studies documented that participation in lymphedema management programs was associated with improved health and well-being, including self-reported quality of life, lower levels of perceived disability and depression, and increased capacity to work.

Improved hygiene and basic skin care were core features of all the intervention packages that were assessed in the studies we reviewed. However, the components of lymphedema management are not fully standardized across LF-endemic areas. This represents not only a limitation of our study but also a challenge to MMDP as key element of the global LF elimination programs. Investigators included movement, leg elevation, and local (e.g., Ayruvedic) practices to varying degrees. Further, lymphedema management programs in filariasis-endemic areas typically have not emphasized the use of emollients to maintain skin hydration and barrier function, although this is recommended by lymphologists and dermatologists in other settings [53].

Our review was also limited by variation in the way morbidity was measured across studies, which made it impossible to conduct meta-analyses for some outcomes. Inconsistent definitions and methods may have introduced error into the meta-analyses. Study design and setting varied, and heterogeneity was high for both meta-analyses on ADLA outcomes, although funnel plots did not appear to show high publication bias (S4 Text).

If basic lymphedema care were provided to all 14 million affected persons in LF-endemic areas, as recommended by WHO [5, 6], considerable benefits would be realized in health, quality of life, and economic productivity. Our meta-analysis suggests, conservatively, that basic lymphedema care could result in at least 14 million fewer ADLA episodes annually (an average of one episode fewer per affected person), marked improvement in quality of life, and perhaps a 10% increase in the number of days worked.

Given these significant benefits and huge burden of disease, it is crucial that LF elimination programs implement lymphedema management. LF program managers have been under considerable pressure to scale up antifilarial drug distribution to interrupt transmission by 2020. In this context, MMDP was often seen as in competition with mass drug treatment. This need not be the case. In fact, providing lymphedema management to affected persons increases acceptance of mass treatment with antifilarial drugs, resulting in higher drug coverage [54]. Further, 2020 is fast approaching and significant LF-related morbidity remains, which threatens the goal of “elimination as a public health problem.” The necessity of MMDP can no longer be ignored. Recent work by WHO to highlight the importance of MMDP and to include indicators for MMDP in criteria for national verification of LF elimination represents important new developments. It is our hope that this review will serve to document the benefits that can be realized through relatively simple hygiene-based measures and thereby encourage their broader application.

During the past few years, the importance of WASH for the control and prevention of major NTDs has been increasingly recognized [55]. Recent systematic reviews highlight the impact of WASH on STH [20], schistosomiasis [21] and trachoma [19]. The current study underscores the crucial importance of WASH—particularly hygiene—in the secondary prevention of morbidity and disability for LF-related lymphedema. The basic hygiene-based interventions recommended for management of lymphedema are also effective for leprosy, Buruli ulcer, and diabetic foot, as well as other chronic conditions of the lower limbs [6, 56]. Further, essentially the same hygiene-based measures are recommended for persons with podoconiosis [57]. Thus, integrated hygiene-based interventions are increasingly recognized as critical for the management of a wide range of lower limb conditions, several of which are diseases of neglected populations, associated with poverty, and targeted for elimination as public health problems. Expansion of hygiene-based measures for morbidity management in resource-poor settings can therefore benefit many more persons than the 14 million affected by LF-associated lymphedema. Refinement of integrated intervention packages and delivery mechanisms is the subject of ongoing research and programmatic innovation. Integrated guidelines that are applicable for LF-related lymphedema, leprosy, podoconiosis, and a range of other diseases have recently been published [58].

In addition to the importance of hygiene in LF-related morbidity management, the other components of WASH—water and sanitation—have played significant roles in preventing the transmission of LF. Improved sanitation and water management can decrease the breeding habitat of mosquitoes, which are responsible for transmitting the parasitic worms that cause LF. Installation of a municipal sewer system was apparently the major factor that led to the elimination of LF transmission in Charleston, South Carolina, where some 29% percent of the population had been infected [59]. Water management, particularly the reduction of standing water, has been crucial in decreasing transmission of LF in other areas [60, 61].

In conclusion, WASH is emerging as a critical factor for the control and elimination of several major NTDs. Recent reviews highlight the importance of WASH in reducing transmission of schistosomiasis, soil-transmitted helminthiasis, and trachoma. Our review underscores the value of WASH in preventing morbidity and promoting healing among persons suffering from LF-associated lymphedema. It also indicates that massive reductions in acute suffering associated with ADLA are possible through improved hygiene-based lymphedema management, and point to significant improvements in quality of life, emotional well-being, and economic productivity. Available data strongly support WHO recommendations to fully realize the intention of the 1997 World Health Assembly resolution 50.29, which called for LF elimination as a public health problem—not only as a parasitic infection.

## Supporting Information

S1 Text PRISMA Checklist. (DOC)

S2 Text Systematic Review Search Protocol Proposal. (DOC)

S3 Text Studies with GRADE scores. (XLSX)

S4 Text Funnel Plots for Publication Bias. (XLSX)
