# Supplementary material for: The Effect of Hygiene-Based Lymphedema Management in Lymphatic Filariasis-Endemic Areas: A Systematic Review and Meta-analysis
Source: PLoS Negl Trop Dis. 2015 Oct 23;9(10):e0004171. doi: 10.1371/journal.pntd.0004171 (PMC4619803; doi:10.1371/journal.pntd.0004171)
Supplement: S2 Text — (DOC) [file pntd.0004171.s002.doc]

**Proposed search protocol for systematic review to evaluate hygiene-related morbidity management programs for lymphatic filariasis**

**Objective**

To examine the effect of hygiene-related morbidity management programs on lymphatic filariasis (LF) morbidity.

**Reporting**

We will adhere to the PRISMA (Preferred Reporting Items for Systematic Reviews and Meta-Analyses) statement.

**Search Methods**

We will systematically PubMed, Embase, ISI Web of Knowledge, MedCarib, Lilacs, REPIDISCA, DESASTRES, and African Index Medicus databases for relevant articles. We will not restrict results based on language or publication date. Our search will be performed until March 23, 2015.

Our database search will include the terms [lymphedema] and [lymphoedema] in combination with the following keywords: [water], [hygiene], [hand wash*], [foot wash*], [soap], [morbidity management], [morbidity control], [disability prevention]

Note: An asterisk (*) denotes a wildcard character.

**Eligibility**

*Inclusion criteria*

Article eligibility will be determined by three key criteria. Studies must contain:

1. Clearly described hygiene-related morbidity management program(s)
2. A direct measurement of LF morbidity (e.g. incidence of ADLA, lymphedema stage or grade, self-reported quality of life score)
3. A distinct parameterization of WASH-related morbidity control program(s) on lymphedema-related morbidity (e.g. incidence rate ratio)

*Study type and participants*

All study types will be eligible for inclusion. There will be no restrictions on setting or study population (e.g. certain age groups or other high-risk populations).

*Hygiene-related morbidity management*

The definition of hygiene-related morbidity management programs will be broad. Data collected through direct observation and through questionnaires/interviews will be eligible for inclusion.

*Outcome measures*

The incidence rate ratios (IRRs) describing the effect of hygiene-related morbidity control programs on incidence rate of ADLA will serve as effect measures in our meta-analysis. We will collect both crude and adjusted estimates where available. If IRRs are not reported, they will be calculated from data provided in articles; if insufficient data is provided within the text, authors will be contacted for additional information (e.g. raw data provided in 2x2 tables). For the purposes of comparison, IRRs will be standardized so that an IRR less than 1.0 indicates a decrease in the incidence rate of ADLA after participating in hygiene-related morbidity management programs.

**Data Collection**

Two reviewers (MES, MP) will independently examine titles and abstracts to determine the relevance of each article. Reviewers will base final selection on the full text of potentially relevant articles. A third reviewer (MCF) will be consulted to determine eligibility of articles in cases of disagreement.

Relevant data from all eligible studies will be collected by one reviewer (MS) into a designated spreadsheet. Extracted data will include:

- Study description (e.g. timeframe, study design, setting, sample size)
- Characteristics of the study population (e.g. age group, socioeconomic details)
- The selection process (e.g. random selection)
- Details about morbidity control program(s) and how data was collected (e.g. foot washing observed by research team, self-reported use of soap)
- Details on how morbidity is measured (e.g. how ADLA is defined, standardized grading system used for staging limbs, details about self-reported quality of life)
- Any other relevant information (e.g. specific strengths or limitations mentioned by authors)
